# Assessment of Cognitive Impairment in Rheumatoid Arthritis

**DOI:** 10.31138/mjr.290724.sah

**Published:** 2025-05-14

**Authors:** Olfa Saidane, Khaoula Zouaoui, Selma Bouden, Leila Rouached, Rawdha Tekaya, Ines Mahmoud, Aicha Ben Tekaya, Leila Abdelmoula

**Affiliations:** 1 Department of Rheumatology, Charles Nicolle Hospital, Tunis, Tunisia;; 2 Department of Rheumatology, La Rabta Hospital, Tunis, Tunisia;; 3 University of Tunis El Manar, Tunisia

**Keywords:** rheumatoid arthritis, cognitive impairment, assessment, cognitive dysfunction

## Abstract

**Objective:**

The objectives of our study were to assess the prevalence of cognitive impairment in Rheumatoid arthritis (RA) and to identify its predictive factors.

**Methods:**

A 6-month cross-sectional case-control study englobing patients with RA was carried out. The cognitive evaluation was performed using the Mini Mental State Examination (MMSE), the frontal efficiency battery, the 5-word test, the clock drawing test and the Trail Making Test part-A (TMT-A). Linear regression analyses were conducted to identify predictors of cognitive impairment.

**Results:**

We included 35 RA patients and 35 controls. Concerning the RA group, the mean duration of the disease was 12.3 years [1–29 years]. RA was immunopositive in 80% of cases and erosive in 83% of cases. The global cognitive dysfunction assessed by MMSE score was 49%. Depending on the test used, the prevalence of cognitive impairment in RA ranged from 34% to 54%. RA patients presented poorer results regarding the TMT-A than the controls (p = 0.03). The other cognitive tests were comparable between the 2 groups. The main predictive independent factors of cognitive impairment among RA patients were advanced age (p = 0.002), rural environment (p = 0.007), low income (p = 0.01), recent course of RA (p = 0.006), low disease activity (p = 0.002) and low blood sugar (p = 0.003).

**Conclusion:**

Global cognitive impairment in RA concerned 49% of our patients. Early identification of the factors associated with this cognitive dysfunction is necessary in order to improve the quality of life of patients.

## INTRODUCTION

Rheumatoid arthritis (RA) is a common chronic inflammatory disease affecting 0.5 to 1% of the adult population^1–3^ characterised by synovial inflammation leading to pain, deformities and joint destruction.^4^

Cognitive functions are defined by the intellectual faculties allowing the acquisition, use, and maintenance of knowledge.^5^

For people with chronic diseases including RA, intact cognitive functions are essential for adequate management of daily activities as well as medication adherence.^6^ It is currently admitted that RA is associated with cognitive impairment. Studies have in fact suggested that more than 30% of RA patients might experience cognitive impairment.^7,8^ This cognitive decline could affect more particularly memory, visuo-spatial function and executive tasks.^9^

The underlying mechanisms of this cognitive impairment are not completely understood. As in the general population, systemic inflammation, and cardiovascular diseases might be linked to cognitive impairment in RA.^8,10–12^ In addition, glucocorticoids, a commonly prescribed medication in RA, could be a risk factor for the genesis of a cognitive impairment.^4^ Depression, frequently encountered in RA, as well as chronic pain also appear to have a significant impact in this cognitive decline.^13^

The prevalence of cognitive impairment in RA and its potential risk factors are unknown in the Tunisian population. Hence, we aimed with this study to assess the prevalence of cognitive impairment in a population of RA, and to identify the factors associated with this cognitive decline.

## MATERIALS AND METHODS

We carried out a single-centre, cross-sectional, case-control study composed of patients followed for RA and healthy controls, in a Tunisian rheumatology department. The inclusion period lasted from September 2018 to March 2019.

The study included patients aged ≥ 18 years, meeting the ACR EULAR 2010 RA criteria^14,15^ who agreed to participate.

The control group was recruited from the patients followed for degenerative rheumatic diseases, patients’ siblings and the paramedical staff of the department. This group was matched to the RA patients group with age, gender and educational level. Patients diagnosed with a neurological and/or psychiatric illnesses, as well as pregnant patients, were excluded from this study. Data collection was identical for all participants, performed by a single examiner (a rheumatologist) in a face-to-face interview for both study groups.

The research protocol was conducted according to the guidelines set in the Helsinki Declaration and was approved by the local ethics committee. All subjects gave their informed consent to participate.

Socio-demographic data were recorded for the 2 groups including sex, age, educational level, marital status, profession, financial income (low, middle, or high income based on local average monthly income) and living environment (rural or urban). For the RA group, we collected information on disease duration, and cardiovascular comorbidities. Serum rheumatoid factor (RF), anticitrullinated antibodies (ACPA), Erythrocyte sedimentation rate (ESR) and C-reactive protein (CRP)levels were also investigated as well as blood sugar. Disease activity was assessed using the Disease Activity Score (DAS28-CRP and DAS28-ESR).^16^ The existence of structural damage was notified based on X-ray and/or ultrasound findings. We also recorded therapeutic data including use of oral glucocorticoids, disease-modifying antirheumatic drugs (DMARDs) and biological therapy. We used the Hospital Anxiety and Depression Scale (HAD) to assess symptoms of depression and/or anxiety.^17^

Both groups underwent a battery of cognitive tests, which included the Mini Mental State Examination (MMSE), the frontal efficiency battery (FAB), the 5-word test (5WT), the clock drawing test (CDT), and the Trail Making Test part-A (TMT-A).

MMSE test^18^ was validated as a screening tool for dementia in the general population and more particularly in the geriatric population. It was translated and adapted to several languages and cultures. In our study, we used the Tunisian version (A-MMSE). The MMSE includes 30 items that explore 6 cognitive fields (orientation, registration, attention, word recall, language, and constructive praxis). The A-MMSE is a 30-point test with 10 points for temporospatial orientation, 3 points for registration, 5 points for concentration, 3 points for memory, 8 points for language and 1 point for constructive praxis. We used a cut-off score of 26 to detect cognitive impairment. This score was lowered to 24 for illiterate patients.

The FAB^19^ is a brief and a simple tool designed to evaluate executive function. It explores 6 items: Similarities (conceptualisation), lexical fluency (mental flexibility), motor programming by carrying out Luria’s series,^20^ conflicting instructions (sensitivity to interference), Go and No-Go test^21^ (Inhibitory control), and prehension behaviour. Each domain is scored from 0 to 3 with a total score ranging from 0 to 18 points. We used a cut-off score of 12 to identify dysexecutive impairment, including illiterate subjects, as the FAB in its Tunisian version was adapted even for people with little or no schooling. The 5WT[22] is an easy and simple tool that has been designed and validated to screen for episodic memory disorder. It consists in making the patient learn a list of 5 words and to evaluate their restitution. The total normal score is 10. We also considered the weighted total score, which must be equal to 20. We used the Tunisian version of the test.

We used threshold scores of 9 and 17 for the total score and the weighted total score, respectively, to detect an episodic memory trouble.

The CDT [23] is a 10-point cognitive instrument validated to screen for visuo-spatial and dysexecutive disorders. The patient is asked to draw a clock then he must place the numbers indicating the hours on the dial and represent a specific time. Three or more errors in drawing the clock indicate a visual and spatial disorder.

For illiterates, we kept the same threshold score, as this test was also validated for those with little schooling.

The TMT-A [24] is a sensitive and validated neuropsychological test to evaluate psychomotor speed. The subject has 25 circles on a sheet of paper, numbered from 1 to 25 and he is instructed to draw lines to connect them in ascending order, as quickly as possible, without lifting the pen.

The patient is considered deficient when he/she exceeds 78 seconds for the test. We did not use this test with illiterate patients.

### Statistical analysis

A descriptive statistical analysis was used to assess the samples’ characteristics and clinical variables. The demographic and baseline outcomes including age, gender, socio-economic conditions, disease duration, cardio-vascular comorbidities, CRP level, ESR level, blood sugar, immunopositivity (for RF and/or ACPA), existence of structural damage, DAS28, HAD, therapeutic approaches (corticosteroids, methotrexate, and biotherapy), in addition to cognitive test results (MMSE, FAB, 5WT, TMT-A and CDT), were described as a mean and standard deviation or median and range for continuous data and percentage for categorical data. Chi-square analyses, Mann-Whitney test, and the Pearson correlation coefficient were used to determine whether significant differences existed between the RA group and the controls, regarding age, gender, socio-economic factors, and cognitive outcomes. For the univariate study, the same statistical analyses were carried out to determine the factors associated with cognitive impairment in RA patients englobing age, gender, living environment, marital status, profession, educational level, financial income, disease duration, cardio-vascular comorbidities, existence of structural damage, DAS28, CRP level, ESR level, blood glucose level, immunopositivity, HAD score and treatments used namely corticosteroids, methotrexate, biologics.

In all statistical tests, the significance level was set at 0.05.

Multivariate analysis was done using linear regression and focused on parameters associated with cognitive impairment in RA, for which the p-level of significance was greater than or equal to 0.15 in the univariate analysis.

For the results in multivariate analysis, A p value < 0.05 was considered statistically significant.

Data were entered and analysed using the Statistical Package for the Social Sciences (SPSS) version 18.0.

## RESULTS

A total of 70 participants were enrolled including 35 RA patients and 35 controls. Both groups were comparable regarding age, gender, and socio-economic conditions. Demographic characteristics are reported in **Table 1**. **Table 2** summarises RA patients’ outcomes. Forty-nine per cent of the RA patients were diagnosed as cognitively impaired according to MMSE. Compared with controls, RA patients had more psychomotor speed troubles detected by TMT-A (43% of patients vs 31% of controls, p= 0.03). Nevertheless, the other cognitive tests were comparable between patients and controls (**Table 3**).

**Table 1.** Demographic data of RA patients and controls at baseline.

		**RA Patients**	**Controls**	**P**
**Age**		**52,1 ± 10,3 [31–74]**	**53,9 ± 10,5 [34–74]**	**0,47**
**Sex**	Male Female	7 28	7 28	1
**Living environment**	Rural environment Urban environment	12 23	7 28	0,17
**Marital status**	Single Married Divorced widower	7 27 0 1	1 32 1 1	0,11
**Profession**	employed unemployed Retired	12 18 5	17 11 7	0,23
**Educational level**	illiterate primary school High school University	2 19 9 5	0 21 10 4	0,52
**Financial income**	Low middle high	11 18 6	13 14 8	0,62

RA: rheumatoid arthritis.

**Table 2.** Patient characteristics^a^.

**Data**	**Mean ± SD / percentage**
Duration of the disease	12,3 ± 8,3 years [1–29 years]
Age of onset of RA	39,8 ± 10,3 years [19–66]
Presence of cardiovascular risk factors	63%
CRP level	10.97 mg/l [0.7–128.5]
ESR	44.62 mm [0–101]
Blood sugar level	5.7 mmol/l [3.98 11.93]
Immunopositivity rate (RF and/or ACPA)	80%
Existence of structural damage	83%
DAS28 (CRP)	4,2 ± 1,4 [1,9–7,4]
DAS28(ESR)	4,8 ± 1,5 [1,4–7,9]
HAD-A score	6.34 ± 6.06 [0–21]
HAD-D score	7.2 ± 6.66 [0–21]
Corticosteroid dose	6.5 ± 5 mg [0–25]
Methotrexate use	69%
Biotherapy use	21%

a: patient characteristics are summarised as mean (SD) [min, max] for continuous variables or frequency (%) for categorical variables

SD: Standard deviation; CRP: C-reactive protein; ESR: Erythrocyte sedimentation rate; RF: Rheumatoid factor; ACPA: anticitrullinated antibodies; HAD: Hospital Anxiety and Depression Scale.

**Table 3.** Comparative study of cognitive impairment.

**Cognitive impairment**	**MMSE**	**FAB**	**5WT**	**Weighted 5WT**	**CDT**	**TMT-A**
**RA patients**	49%	34%	49%	51%	54%	43%
**Controls**	34%	29%	37%	40%	47%	31%
**p**	0.22	0.60	0.33	0.33	0.54	0.03

MMSE: Mini Mental State Examination; FAB: Frontal efficiency battery; 5WT: five word-test; CDT: clock drawing test; TMT: trail making test part A; RA: rheumatoid arthritis.

In subgroup analysis, males experienced more memory dysfunction than women (p=0.003 for the 5WT and p=0.008 for the weighted 5WT). Additionally, cardio-vascular risk factors were associated with decline of episodic memory (p=0.02). Interestingly, patients starting RA at a later age were more likely to develop cognitive impairment as shown by the FAB, the 5-word test (total and weighted) and the clock drawing test (p=0.02, r=−0.370; p=0.01, r=−0.432; p=0.04, r=−0.348; p=0.03, r=−0.359 respectively).

Predictive factors of cognitive impairment in RA, revealed by linear regression were advanced age, rural environment, low income, recent evolution of the disease, low DAS28 (ESR) and low blood sugar (**Table 4**).

**Table 4.** Predictive factors of cognitive impairment in RA.

**Risk factor**	**p**	**B**	**Cognitive test**
Males	0.527	1.694	Total 5WT
Advanced age	0.002* 0.003*	−0.071 −0.071	total 5WT weighted 5WT
Rural environment	0.007* 0.006*	−1.183 −1.247	total 5WT weighted 5WT
Low Income	0.011*	0.677	weighted 5WT
Cardio-vascular risk factors	0.925	−0.029	Total 5WT
Recent evolution of the disease	0.006*	0.263	CDT
Age at onset of RA	0.851	0.017	CDT
Low DAS28(ESR)	0.002*	1.496	CDT
Low blood sugar	0.003*	1.160	CDT

B: linear regression coefficient; 5WT: five word-test; CDT: clock drawing test.

*: significant results.

## DISCUSSION

This cross-sectional study showed that prevalence of cognitive impairment ranged from 34% to 54% in RA patients, depending on the assessing tool used. The cognitive impairment affected predominantly psychomotor speed, in addition to memory and executive functions.

These finding were similar to previous studies investigating cognitive disturbance in patients with RA.^7,8,25,26^ Thus, Bartolini et al. noticed a prevalence of cognitive impairment varying from 38% for attention and mental flexibility to 71% for visual-spatial ability and planning.^25^ An American study conducted by Shin et al*.* identified a cognitive impairment in 31% of RA patients^8^ with more than 20% of patients suffering from executive function and visuospatial ability impairment and 18% of patients from memory disorders. In the Vitturi et al. study, up to 74.5% and 97.6% of RA patients had cognitive impairment detected by the MMSE and the Montreal Cognitive Assessment (MoCA) respectively.^4^

Assessing cognitive impairment during RA is challenging. A substantial number of assessment instruments had been used in the literature.^27^ Additionally, the absence of standardised cognitive tests developed and validated for RA^8^ and the heterogeneity of the populations studied might have contributed to the discrepancy in results between studies. In our work, we used reproductible cognitive screening tools, simple to administer to participants, and validated in many chronic diseases.^18,19,22–24^

The influence of age on cognitive impairment in RA patients seems controversial.

An American study published in 2021 found no association between cognitive impairment and rheumatoid arthritis in older patients.^28^ Conversely, according to Vitturi et al., and in line with our study, advanced age was a non-modifiable demographic factor impacting cognitive function, and more specifically memory of RA patients.^4^ Meade et al. had reported that age was likewise associated with psychomotor speed disorders sought by the trail making test.^29^ This could be explained by the importance of associated comorbidities and cardiovascular risk factors.^4^ Another possible explanation would be the relationship with normal cognitive aging.^30^ However, cognitive impairment was also observed in young RA patients, especially during the early stages of the disease.^9^ This cognitive impairment, occurring early in the course of the disease, would be attributable to the pathogenesis of RA itself which is distinguished by a significant inflammatory process.^9^ In line with these findings, we noted that patients with a recent RA were at a greater risk of developing visuospatial dysfunction.

The role of socio-economic conditions in the development of cognitive impairment in RA patients has been mentioned in certain studies.^8,9,31,32^ Shin et al. found a significant association between low income and cognitive impairment.^8^ In the current study, a low income was found to be a predictive factor of cognitive impairment in patients. A Greek study conducted by Simos et al*.* noticed no significant link between the living area and cognitive impairment in RA.^9^ Nevertheless, we reported that patients living in rural areas were more likely to develop cognitive dysfunction.

Cognitive impairment might be associated with disease activity.^33^ Notably, many studies found an association between DAS28 and cognitive dysfunction.^34^ Particularly, in the study of Katchamart et al. involving 464 RA patients, disease activity was significantly associated with cognitive impairment.^6^ It is hypothesised that systemic inflammation related to rheumatic diseases along with chronic pain might be the cause of the cognitive impairment.^34^ Paradoxically, in our work, low disease activity parameters were associated with worse cognitive outcomes.

To our knowledge, no studies have investigated the direct role of blood glucose in the cognitive function of RA patients. In the current work, we found that a low blood sugar was a predictive factor of visuospatial disorders. This might be explained by the importance of glucose for adequate brain function and therefore for maintaining correct cognitive faculties.^35^

Other factors were found to be associated with cognitive decline in the literature although this was not observed in this current study.

Among psychiatric conditions, anxiety and depression seem to be linked to cognitive impairment during RA with a negative influence on pain, well-being and quality of life.^34^

Cardiovascular risk factors were also found to be involved in the genesis of cognitive disorders^36^ as shown by the study of Shin et al.^8^ In our study, and in agreement with these data, the univariate analysis showed that the existence of cardiovascular risk factors was associated with memory disturbances, but we did not find such results in the multivariate analysis.

RA therapeutics might influence cognitive function in RA patients. Notably, the use of glucocorticoids was found to be a predictive factor of cognitive impairment in RA patients in the study of Shin et al.^8^ Conversely, methotrexate and TNF blockers in RA might be protective by reducing the risk of dementia.^26,34,37^

Our study has some limitations. The sample size could add some weaknesses to our results. Additionally, we conducted a single-centre cross-sectional study. The lack of a longitudinal follow-up is another limitation of this study. Also, the cognitive assessment was not carried out using extensive batteries of neuropsychological tests, which could constitute an information bias. Thus, larger multicentric cohort studies are needed for a more accurate assessment of cognitive impairment during RA and its risk factors. A better understanding of these risk factors could possibly lead to new prognostic and therapeutic approaches.

## CONCLUSION

In this study, we found that cognitive impairment is common in rheumatoid arthritis. Advanced age, unfavourable socio-economic conditions and a recent course of the disease are potentially predictive factors of this cognitive impairment. Closely monitor cardiovascular risk factors and inflammation might help to prevent or to slow cognitive decline.

## CONFLICT OF INTEREST

All authors have no conflicts of interest to declare.

## FUNDING

There has been no financial support for this work that could have influenced its outcomes.

## ETHICS APPROVAL AND CONSENT TO PARTICIPATE

The research protocol was conducted according to the guidelines set in the Helsinki Declaration and was approved by the local ethics committee of la Rabta Hospital. All subjects gave their informed consent to participate.

## References

[1] Gibofsky A. Epidemiology, Pathophysiology, and Diagnosis of Rheumatoid Arthritis: A Synopsis. Am J Manag Care 2014 May;20(7 Suppl):S128–35 [cited 2023 Apr 24];20. Available from: https://www.ajmc.com/view/ace017_may14_ra-ce_gibofsky1_s128 25180621

[2] Alamanos Y Drosos AA. Epidemiology of adult rheumatoid arthritis. Autoimmun Rev 2005 Mar;4(3):130–6. [cited 2023 Aug 17]. Available from: https://pubmed.ncbi.nlm.nih.gov/15823498/15823498 10.1016/j.autrev.2004.09.002

[3] Gabriel SE Michaud K. Epidemiological studies in incidence, prevalence, mortality, and comorbidity of the rheumatic diseases. Arthritis Res Ther 2009;11(3):229. 19519924 10.1186/ar2669 PMC2714099

[4] Vitturi BK Nascimento BAC Alves BR de Campos FSC Torigoe DY. Cognitive impairment in patients with rheumatoid arthritis. J Clin Neurosci 2019 Nov;69:81–7. 31447371 10.1016/j.jocn.2019.08.027

[5] Fontana IL. Impact du style de vie sur le vieillissement cognitif: Étude des modérateurs du déclin cognitif tout au long de la vie adulte. Comment les différences hommes/femmes amènent à reconsidérer l’influence du style de vie sur le fonctionnement cognitif? Psychologie 2017, Université Paris Saclay (COmUE). [Français]

[6] Katchamart W Narongroeknawin P Phutthinart N Srinonprasert V Muangpaisan W Chaiamnauy S. Disease activity is associated with cognitive impairment in patients with rheumatoid arthritis. Clin Rheumatol 2019 Jul;38(7):1851–6. 30848400 10.1007/s10067-019-04488-3

[7] Appenzeller S Bertolo MB Costallat LTL. Cognitive impairment in rheumatoid arthritis. Methods Find Exp Clin Pharmacol 2004 Jun;26(5):339–43. 15319812 10.1358/mf.2004.26.5.831324

[8] Shin SY Katz P Wallhagen M Julian L. Cognitive impairment in persons with rheumatoid arthritis. Arthritis Care Res 2012;n/a-n/a. 10.1002/acr.21683 PMC3744877 22505279

[9] Simos P Ktistaki G Dimitraki G Papastefanakis E Kougkas N Fanouriakis A Cognitive deficits early in the course of rheumatoid arthritis. J Clin Exp Neuropsychol 2016 Aug 8;38(7):820–9. 27133019 10.1080/13803395.2016.1167173

[10] Hashimoto H Kawamura M Yukami T Ishihara M Bamba Y Kaneshiro S Etiology of acute ischaemic cerebrovascular disease associated with rheumatoid arthritis: changes with progression of anti-inflammatory therapy. Eur J Neurol 2018 Dec;25(12):1462–9. 29995999 10.1111/ene.13751

[11] Meade T Manolios N Cumming SR Conaghan PG Katz P. Cognitive Impairment in Rheumatoid Arthritis: A Systematic Review. Arthritis Care Res 2018 Jan;70(1):39–52. 10.1002/acr.23243 28371512

[12] Dregan A Chowienczyk P Gulliford MC. Are Inflammation and Related Therapy Associated with All-Cause Dementia in a Primary Care Population? Caruso C, editor. J Alzheimers Dis 2015 Jun 26;46(4):1039–47. 26402631 10.3233/JAD-150171

[13] Brown SC Glass JM Park DC. The relationship of pain and depression to cognitive function in rheumatoid arthritis patients. Pain 2002 Apr;96(3):279–84. 11973000 10.1016/S0304-3959(01)00457-2

[14] Arnett FC Edworthy SM Bloch DA Mcshane DJ Fries JF Cooper NS The American Rheumatism Association 1987 revised criteria for the classification of rheumatoid arthritis. Arthritis Rheum 1988 Mar;31(3):315–24. 3358796 10.1002/art.1780310302

[15] Aletaha D Neogi T Silman AJ Funovits J Felson DT Bingham CO 2010 Rheumatoid arthritis classification criteria: An American College of Rheumatology/European League Against Rheumatism collaborative initiative. Arthritis Rheum 2010 Sep;62(9):2569–81. 20872595 10.1002/art.27584

[16] Prevoo ML van’t Hof MA Kuper HH van Leeuwen MA van de Putte LB van Riel PL. Modified disease activity scores that include twenty-eight-joint counts. Development and validation in a prospective longitudinal study of patients with rheumatoid arthritis. Arthritis Rheum 1995 Jan;38(1):44–8. 7818570 10.1002/art.1780380107

[17] Terkawi AS Tsang S AlKahtani GJ Al-Mousa SH Al Musaed S AlZoraigi US Development and validation of Arabic version of the Hospital Anxiety and Depression Scale. Saudi J Anaesth 2017 May;11(Suppl 1):S11–8. 28616000 10.4103/sja.SJA_43_17 PMC5463562

[18] Bellaj T Jemaa SB Romdhane NA Dhiffallah M Ali NB Bouaziz M Version arabe du mini mental state examination (A-MMSE): fidélité, validité et données normatives. Tunis Med 2008;86(7):767–75.PubMed. [cited 2023 Aug 17].

[19] Jemaa SB Bellaj T Romdhane NA Chérif A Zakraoui NO Bouaziz M. La frontal assessment battery (FAB): fidélité, validité et étalonnage d’une forme arabe. Tunis Med. 2008;86(7):793–800. PubMed. [cited 2023 Aug 17].

[20] Weiner MF Hynan LS Rossetti H Falkowski J. Luria’s three-step test: what is it and what does it tell us? Int Psychogeriatr 2011 Dec;23(10):1602–6. 21554794 10.1017/S1041610211000767 PMC3399685

[21] Gomez P Ratcliff R Perea M. A model of the go/no-go task. J Exp Psychol Gen 2007 Aug;136(3):389–413. 17696690 10.1037/0096-3445.136.3.389 PMC2701630

[22] Khiari HM Romdhane NA Bellaj T Bennys K Anane N Mrabet A. Version arabe de l’épreuve des 5 mots : validation clinique pour le diagnostic de démence de type alzheimer. Tunis Med. 2008;86(7):786–92. [cited 2023 Aug 17]. Available from:

[23] Shulman KI. Clock-drawing: is it the ideal cognitive screening test? Int J Geriatr Psychiatry 2000 Jun;15(6):548–61. 10861923 10.1002/1099-1166(200006)15:6<548::aid-gps242>3.0.co;2-u

[24] Bowie CR Harvey PD. Administration and interpretation of the Trail Making Test. Nat Protoc 2006;1(5):2277–81. 17406468 10.1038/nprot.2006.390

[25] Bartolini M Candela M Brugni M Catena L Mari F Pomponio G Are behaviour and motor performances of rheumatoid arthritis patients influenced by subclinical cognitive impairments? A clinical and neuroimaging study. Clin Exp Rheumatol 2002;20(4):491–7. 12175104

[26] McDowell B Marr C Holmes C Edwards CJ Cardwell C McHenry M Prevalence of cognitive impairment in patients with rheumatoid arthritis: a cross sectional study. BMC Psychiatry 2022 Dec 9;22(1):777. 36494656 10.1186/s12888-022-04417-w PMC9733399

[27] Pankowski D Wytrychiewicz-Pankowska K Janowski K Pisula E. Cognitive impairment in patients with rheumatoid arthritis: A systematic review and meta-analysis. Joint Bone Spine 2022 May;89(3):105298. 34656753 10.1016/j.jbspin.2021.105298

[28] Booth MJ Janevic MR Kobayashi LC Clauw DJ Piette JD. No association between rheumatoid arthritis and cognitive impairment in a cross-sectional national sample of older U.S. adults. BMC Rheumatol. 2021 Dec;5(1):24. 34404491 10.1186/s41927-021-00198-z PMC8371766

[29] Meade T Cumming S Hallab L Spencer D Howe G Manolios N. A preliminary investigation of cognitive function in rheumatoid arthritis patients on long-term methotrexate treatment. J Health Psychol 2013 Oct;18(10):1353–9. 23188915 10.1177/1359105312461660

[30] Kramer AF Erickson KI Colcombe SJ. Exercise, cognition, and the aging brain. J Appl Physiol Bethesda Md 1985 2006 Oct;101(4):1237–42. 10.1152/japplphysiol.00500.2006 16778001

[31] Shin SY Katz P Julian L. Relationship between perceived cognitive dysfunction and objective neuropsychological performance in persons with rheumatoid arthritis. Arthritis Care Res 2013 Mar;65(3):481–6. 10.1002/acr.21814 PMC3786333 22899659

[32] Yoon BY Lee JH Shin SY. Discrepancy between subjective and objective measures of cognitive impairment in patients with rheumatoid arthritis. Rheumatol Int 2017 Oct;37(10):1635–41. 28871379 10.1007/s00296-017-3806-2

[33] Lee JH Kim GT Kim YK Lee SG. Cognitive function of patients with rheumatoid arthritis is associated with disease activity but not carotid atherosclerotic changes. Clin Exp Rheumatol 2018;36(5):856–61. 29652660

[34] Basile MS Ciurleo R Bramanti A Petralia MC Fagone P Nicoletti F Cognitive Decline in Rheumatoid Arthritis: Insight into the Molecular Pathogenetic Mechanisms. Int J Mol Sci 2021 Jan 26;22(3):1185. 33530359 10.3390/ijms22031185 PMC7865873

[35] Shen X Liu H Hu Z Hu H Shi P. The relationship between cerebral glucose metabolism and age: report of a large brain PET data set. PloS One 2012;7(12):e51517. 23284706 10.1371/journal.pone.0051517 PMC3527454

[36] Sahathevan R Brodtmann A Donnan GA. Dementia, stroke, and vascular risk factors; a review. Int J Stroke Off J Int Stroke Soc 2012 Jan;7(1):61–73. 10.1111/j.1747-4949.2011.00731.x 22188853

[37] Hugon J. Rheumatoid arthritis and cognitive decline. Joint Bone Spine 2022 May;89(3):105346. 35066187 10.1016/j.jbspin.2022.105346

